# Fatty Acid-Binding Protein 5 Gene Deletion Enhances Nicotine-Conditioned Place Preference: Illuminating the Putative Gateway Mechanisms

**DOI:** 10.3390/futurepharmacol3010007

**Published:** 2023-01-09

**Authors:** Nicole Roeder, Brittany Richardson, Abrianna Mihalkovic, Samantha Penman, Olivia White, John Hamilton, Ashim Gupta, Kenneth Blum, Mark S. Gold, Panayotis K. Thanos

**Affiliations:** 1 Behavioral Neuropharmacology and Neuroimaging Laboratory, Clinical Research Institute on Addictions, Department of Pharmacology and Toxicology, Jacobs School of Medicine and Biomedical Sciences, University at Buffalo, Buffalo, NY 14203, USA; 2 Department of Psychology, University at Buffalo, Buffalo, NY 14203, USA; 3 Future Biologics, Lawrenceville, GA 30043, USA; 4 Division of Addiction Research & Education, Center for Mental Health & Sports, Exercise and Global Mental Health, Western University Health Sciences, Pomona, CA 91766, USA; 5 Department of Psychiatry, School of Medicine, University of Vermont, Burlington, VT 05405, USA; 6 Department of Psychiatry, Washington University School of Medicine, St. Louis, MO 63110, USA

**Keywords:** nicotine, conditioned place preference, fatty acid-binding protein 5, endocannabinoids, cannabinoid receptor 1, dopamine

## Abstract

Emerging evidence indicates that the endogenous cannabinoid system modulates the behavioral and physiological effects of nicotine. Fatty acid-binding proteins (FABPs) are among the primary intracellular trafficking mechanisms of endogenous cannabinoids, such as anandamide. To this end, changes in FABP expression may similarly impact the behavioral manifestations associated with nicotine, particularly its addictive properties. *FABP5*^+/+^ and *FABP5*^−/−^ mice were tested for nicotine-conditioned place preference (CPP) at two different doses (0.1 or 0.5 mg/kg). The nicotine-paired chamber was assigned as their least preferred chamber during preconditioning. Following 8 days of conditioning, the mice were injected with either nicotine or saline. The mice were allowed to access to all the chambers on the test day, and their times spent in the drug chamber on the preconditioning versus the test days were used to examine the drug preference score. The CPP results showed that the *FABP5*^−/−^ mice displayed a higher place preference for 0.1 mg/kg nicotine than the *FABP5*^+/+^ mice, while no CPP difference was observed for 0.5 mg/kg nicotine between the genotypes. In conclusion, *FABP5* plays an important role in regulating nicotine place preference. Further research is warranted to identify the precise mechanisms. The results suggest that dysregulated cannabinoid signaling may impact nicotine-seeking behavior.

## 1. Introduction

Cigarette smoking remains a leading preventable cause of death in the United States [1]. Although most of the toxicity of cigarette smoking is related to other components of combustible cigarettes, nicotine is the primary addictive component [2]. When inhaled, nicotine readily infuses into the brain and binds to the nicotinic acetylcholine receptors, releasing neurotransmitters, including dopamine (DA), into the mesolimbic regions of the brain [2]. Specifically, nicotine stimulates the activation of DAergic neurons within the ventral tegmental area (VTA) and the nucleus accumbens (NAc) shell, which are critical regions for the rewarding properties of nicotine [2,3]. Additionally, DAergic signaling is an important player in the transmission of reward-related information [4] and is believed to be the modulator of behaviors associated with nicotine use [5].

The DAergic signaling within the mesolimbic reward pathway is modulated by the endocannabinoid (eCB) system [6,7]. This system is comprised of cannabinoid type-1 and type-2 G-protein-coupled receptors (CB1R and CB2R, respectively) and two major endogenous ligands, anandamide (AEA) and 2-arachidonoyl-glycerol (2-AG), as well as the enzymes involved in their synthesis and metabolism, fatty acid amide hydrolase (FAAH) and monoacylglycerol lipase (MAGL) [8]. The dysfunction of eCB signaling has been implicated in the pathophysiology of psychiatric disorders, such as schizophrenia, substance abuse, depression, and anxiety disorders [9–12]. Cannabinoid receptor signaling is tied to the reinforcing properties associated with nicotine. Rimonabant, a CB1R inverse agonist, has been shown to decrease nicotine self-administration and conditioned place preference in the translational literature [13,14]. This CB1R inverse agonist has also been shown to prolong abstinence rates among smokers who are motivated to quit smoking [15]. The blocking of CB1R activation has also been shown to attenuate nicotine-induced DA increases in the NAc [16–18]. Furthermore, reduced nicotine-seeking behavior in animal models of relapse has been observed with the application of rimonabant [19]. These findings indicate that CB1Rs and eCBs are important for nicotine and the motivation to seek the drug. Kandel and Kandel have published several papers on the potential gateway theories of certain drugs, such as nicotine, acting as a gateway to other drugs of abuse [20].

In addition, other research has examined eCB signaling, such as FAAH, which is involved in the synthesis and metabolism of eCBs. Specifically, FAAH is known to rapidly metabolize AEA, which has been shown to have a high affinity with CB1Rs on the presynaptic neurons that activate the mesolimbic DA system [21]. AEA is synthesized on demand, and upon release, it is quickly degraded by FAAH into arachidonic acid and ethanolamine [21–23]. *FAAH*^−/−^ mice, which have a 10- to 15-fold increase in the brain AEA levels, show enhanced CPP in response to a low dose of nicotine (0.1 mg/kg), whereas their *FAAH*^+/+^ counterparts had no nicotine acquisition [24]. These effects were reversed following the blockade of CB1Rs, indicating that these actions are CB1R-mediated. At higher doses, such as 0.5 and 1 mg/kg nicotine, both the *FAAH*^+/+^ and *FAAH*^−/−^ mice displayed no difference in nicotine CPP. A more recent microdialysis investigation of a dose of 0.1 mg/kg nicotine observed elevated DA concentrations within the NAc of *FAAH*^−/−^ mice [25], thus suggesting that elevated eCB levels enhance the rewarding properties associated with nicotine.

More recently, fatty acid-binding proteins (FABPs) have been described as intracellular chaperone proteins that facilitate the uptake and transport of AEA to FAAH for degradation [26]. Genetic deletion of the *FABP* genes elevates the whole-brain AEA levels [27]. *FABP5*^−/−^ mice have also been shown to have heightened levels of AEA and 2-AG in the midbrain when compared to *FABP5*^+/+^ mice [28]. Previous research has indicated that 2-AG plays a role in glutamate signaling on DA neurons in the VTA, which may play a role in tobacco addiction [29,30]. Given the potential impact of eCB signaling on nicotine reward, the current study sought to determine the specific behavioral effects of the *FABP5* gene in regulating the rewarding effects of nicotine. We hypothesized that because *FABP5* serves as an intracellular carrier of AEA, the deletion of this gene would enhance the rewarding properties of nicotine in *FABP5*^−/−^ mice.

## 2. Materials and Methods

### 2.1. Animals

Male and female *FABP5*^+/+^ and *FABP5*^−/−^ mice on a C57B6 background, as described previously [31], were kindly provided by Dr. Hotamisligil at Harvard University. The mice were bred in-house, as previously described. All the mice were habituated to the holding room for at least one week and tested between 10 and 14 weeks of age. Before testing, the mice were habituated to handling and subcutaneous (sc) injections. All the mice were drug-naive at the start of the testing and single-housed in a temperature-controlled room on a reverse 12 h light/dark cycle (lights off from 0900–1800). The animals were provided with ad libitum access to food and water throughout the experiments. All the experiments and procedures conformed to the National Institutes of Health Guidelines for the Care and Use of Laboratory Animals. The protocol was approved by the Institutional Animal Care and Use Committee at the University of Buffalo, NY, USA.

### 2.2. Drugs

Nicotine hydrogen tartrate salt was purchased from Sigma-Aldrich (St. Louis, MO, USA). On the first day of each experiment, the animal body weights were recorded, and nicotine solutions were prepared by dissolving 0.01 mg of nicotine per 1 mL of saline. Each administered nicotine dose was based on the individual animal body weight, and all the doses were injected via sc injections at 10 mL/kg. On the drug days of the nicotine-conditioned place preference paradigm, the mice were injected (sc) with either 0.1 or 0.5 mg/kg nicotine immediately before being placed in the nicotine-paired conditioning chamber. These doses were based on previous studies of nicotine CPP [24].

### 2.3. Statistical Analysis

All statistical analyses were conducted using GraphPad Prism v. 9.3.0 (GraphPad Software Inc., San Diego, CA, USA). The nicotine-conditioned place preference data analysis was conducted using an unpaired two-tailed *t*-test (comparing differences between the time spent (delta) in the preconditioning versus the test phases, as well as the average locomotor activity per drug dose). The sexes were compared first (males versus females) to determine the potential sex differences, followed by the comparison of the genotypes (*FABP*^+/+^ versus *FABP5*^−/−^).

### 2.4. Nicotine-Conditioned Place Preference (CPP)

The *FABP5*^+/+^ and *FABP5*^−/−^ mice were tested for nicotine CPP using commercially available equipment (Coulbourn Instruments, Allentown, PA, USA). The mice were tested in three phases: preconditioning, conditioning, and the test day, as previously described [32–36]. Briefly, the place-conditioning boxes contained three compartments with distinct flooring and wall patterns for tactile and visual differentiation (black/white polka dots with plexiglass flooring or black/white stripes with metal flooring), which were separated by a neutral clear plexiglass compartment. During the preconditioning phase, movement between the distinct polka or striped compartments was possible through the use of two manual guillotine doors, which provided access to either chamber when opened. The entrances to both chambers were closed during the vehicle and drug conditioning days. During the intertrial intervals, the chambers were wiped clean. The testing took place for a total of 10 days and was conducted between 1200 and 1700 each day during the animal’s dark cycle. The mice were subjected to one of two experiments. Experiment one (Exp 1) tested the mice for nicotine CPP at the 0.1 mg/kg nicotine dose. Experiment two (Exp 2) tested the mice for nicotine CPP at the 0.5 mg/kg nicotine dose. Below is a brief summary of the procedure.

#### Day 1: Preconditioning phase.

The mice were placed in the neutral center chamber and allowed to access the distinct compartments, with the guillotine doors open, for a total of 15 min. The time spent on each side was recorded in seconds and compiled as a percentage of the time spent in each chamber to determine each subject’s baseline place preference. Animals who exhibited an equal preference between chambers were randomized for the following conditioning phase.

#### Days 2 to 9: Conditioning phase.

Both the *FABP5*^+/+^ and *FABP5*^−/−^ mice received sc injections of either saline or nicotine on alternating days and were immediately placed in their respective chambers for the next 8 days of testing. The conditioning took place for 20 min, in which the mice were free to roam the chamber corresponding to the injection received. The drug-paired sides were pre-determined as the opposite of the mice’s initial baseline preference, and the initially preferred chambers were pre-determined as the vehicle treatment (i.e., the biased paradigm). The injections were counterbalanced with respect to the side of the chamber on which the animal was placed. For example, if the animal’s least preferred chamber had the stripped walls and plexiglass flooring during the preconditioning phase, they would be placed in this chamber on the days when they received nicotine injections to measure the difference in their drug-induced place preference later on the test day (day 10). On the saline days, they would be placed in the polka dot chamber with metal flooring, and vice versa. The total number of conditioning days for nicotine and saline was equally divided: four days of nicotine conditioning exposure and four days of saline conditioning exposure.

#### Day 10: Test phase.

On the final day, all the animals were placed in the center neutral compartment without exposure to either saline or nicotine. The guillotine doors were opened, and the subjects were given free-roam access to either chamber for 15 min. The time spent on each side was automatically recorded in seconds, and the mice’s preference for the drug-paired chamber was expressed as a percentage of the time spent on the drug-paired side on the test day (day 10) minus their baseline percentage of time spent in their assigned drug chamber on the preconditioning day (day 1). A positive number indicated a preference for the drug-paired chamber, whereas a negative number indicated aversion. A value of zero indicated no preference for either side.

## 3. Results

### 3.1. Nicotine CPP

In each experiment, the treatment group was tested for their change (delta) in preference to the nicotine-paired chamber by measuring the total time spent (seconds) in the nicotine-paired chamber on the preconditioning day versus the test day. Outlier testing (ROUT, Q = 1%) was completed for both the experiments, and none were observed.

#### Exp 1 (0.1 mg/kg Nicotine CPP):

**Figure 1.**
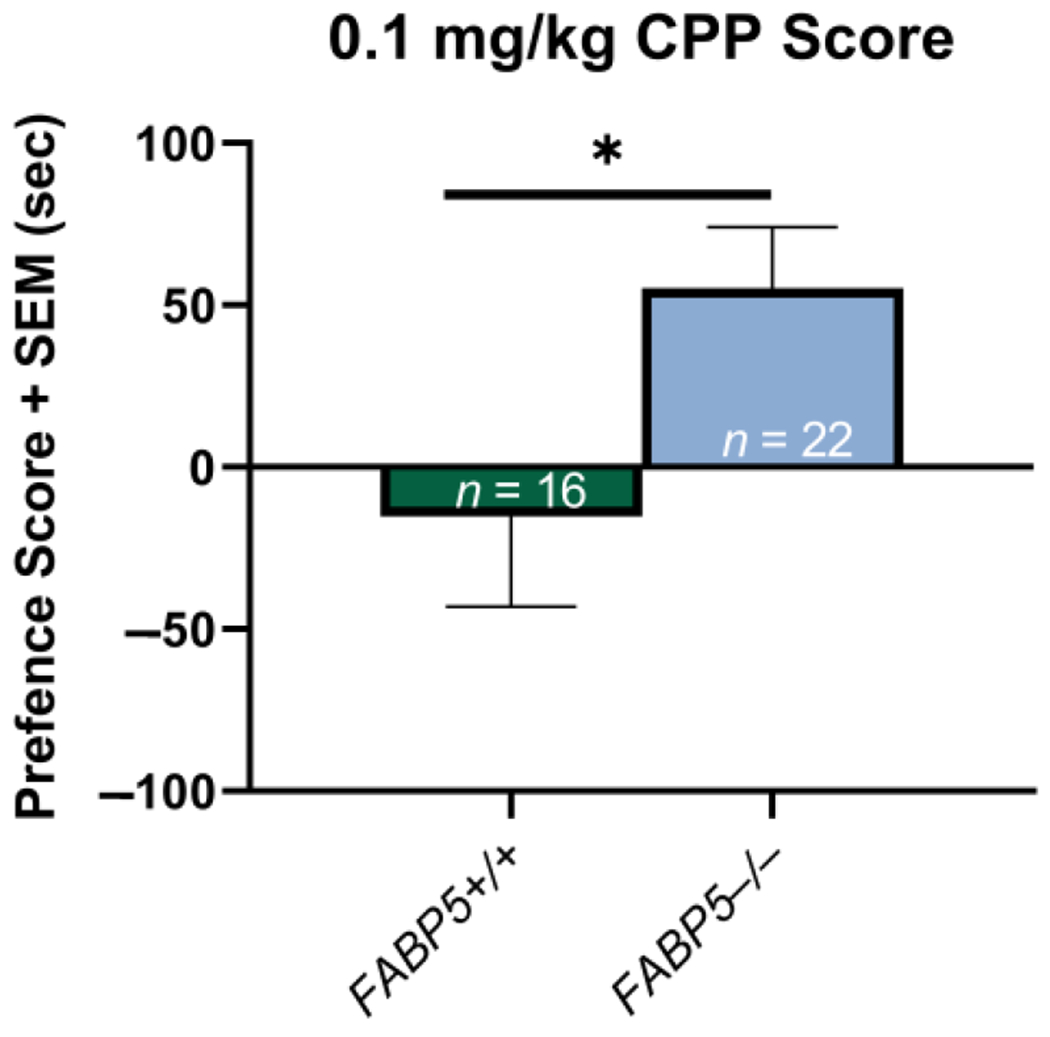
Effects of 0.1 mg/kg nicotine on the conditioned place preference paradigm between the *FABP5*^+/+^ and *FABP5*^−/−^ mice. The CPP scores are defined as the difference between the time spent in the nicotine-paired compartment in the test versus the pretest phases. * *p* < 0.05.

Potential sex differences in the delta preference scores between the *FABP5*^+/+^ and *FABP5*^−/−^ groups were assessed as previously described, and none were observed (*p* > 0.05). The sexes were then collapsed to compare the genotypes within the groups, and the results showed that at the 0.1 mg/kg nicotine dose, the *FABP5*^−/−^ mice had a significantly higher preference score for the nicotine-paired chamber, *t*(35) = 2.18, * *p* = 0.036, compared with their *FABP5*^+/+^ counterparts (see Figure 1).

#### Exp 2 (0.5 mg/kg Nicotine CPP):

**Figure 2.**
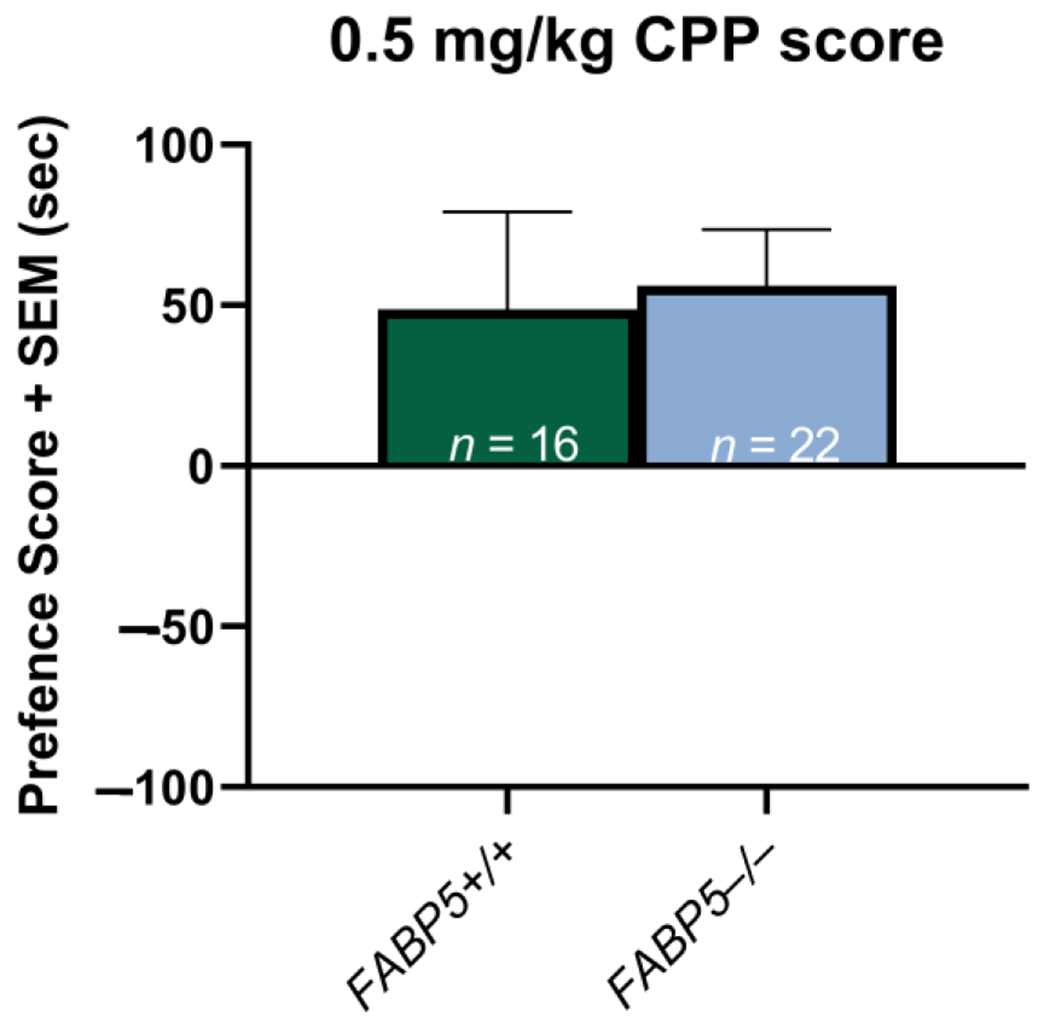
Effects of 0.5 mg/kg nicotine on the conditioned place preference paradigm between the *FABP5*^+/+^ and *FABP5*^−/−^ mice. The CPP scores are defined as the difference between the time spent in the nicotine-paired compartment in the test versus the pretest phases.

Potential sex differences in the delta preference scores between the *FABP5*^+/+^ and *FABP5*^−/−^ groups were assessed as previously described, and none were observed (*p* > 0.05). The sexes were then collapsed to compare the genotypes within the groups, and the results showed no significant difference (*p* > 0.05) between the *FABP5*^−/−^ mice and their *FABP5*^+/+^ counterparts (see Figure 2).

### 3.2. Nicotine CPP Locomotor Activity

Each group’s locomotor activity was assessed for each dose, as measured by the average photobeam breaks on the conditioning days. For both Exp 1 (0.1 mg/kg nicotine CPP) and Exp 2 (0.5 mg/kg nicotine CPP), there was no significant difference (*p* > 0.05) in the locomotor activity when comparing saline injections to the nicotine injections in the case of either genotype.

## 4. Discussion

The present study examined the role of the gene encoding of the endocannabinoid-trafficking protein, FABP5, on nicotine CPP. For the first time, we demonstrated the novel role of this protein in regulating the rewarding properties associated with nicotine. Mice genetically deficient in *FABP5* showed greater acquisition of a nicotine place preference at a low nicotine dose of 0.1 mg/kg (Figure 1). Unlike the *FABP5*^+/+^ mice, the *FABP5*^−/−^ did not show a dose-dependent effect of nicotine CPP. This enhanced CPP acquisition for 0.1 mg/kg nicotine supports our hypothesis regarding what is known about the role of cannabinoids and nicotine reward.

The observed phenotype in the *FABP5*^−/−^ mice, associated with a subthreshold nicotine dose of 0.1 mg/kg, is supported by the findings of Merritt and colleagues, who tested *FAAH*^−/−^ mice and demonstrated the enhanced acquisition of nicotine place preference with the same low dose of nicotine (0.1 mg/kg) but not at the high doses of nicotine (0.5 or 1 mg/kg) [24]. However, the effect on the *FAAH*^−/−^ mice was greater in magnitude compared with the FABP5^−/−^ mice in the current study, which is plausible, since *FAAH*^−/−^ mice show a ~15-fold increase in the AEA levels in the brain, while *FABP5*^−/−^ mice only show a −1.5-fold increase in AEA [37]. Microdialysis studies of *FAAH*^−/−^ mice indicated that 0.1 mg/kg of nicotine significantly increases the DA concentrations in the NAc [25], which may underlie the enhanced place preference for nicotine in CPP paradigms. A similar mechanism of action may underlie the behavior observed in *FABP5*^−/−^ mice, though this has yet to be evaluated.

Additionally, it is also possible that the enhanced nicotine CPP acquisition observed in *FABP5*^−/−^ mice could be due not only to the AEA levels but also to heightened 2-AG levels. Heightened 2-AG levels have been reported in the midbrain of *FABP5*^−/−^ mice when compared to their *FABP5*^+/+^ counterparts [28], including areas such as the VTA. Previous research has determined that nicotine increases the VTA dialysate 2-AG levels under conditions of acute and chronic administration [30]. It was found that 2-AG plays a key role in the plasticity of glutamate signaling to DA neurons in the VTA [38], which may be a critical component of the mechanisms of tobacco addiction [39]. When there are higher levels of 2-AG in the VTA, a cue-evoked increase in DA within the NAc is potentiated, which has been associated with reward-seeking behavior [40]. In addition, VTA AEA signaling may enhance DA cell activity through CB1R-mediated decreases in GABA release [41,42]. Indeed, the CB1R-mediated suppression of VTA glutamate release has also been reported [41,43], which may contribute to the CB1R-mediated attenuation of the nicotine-induced excitation of DA cells in the VTA following FAAH inhibition [44]. The global deletion of the *FABP5* gene significantly decreased tonic 2-AG and AEA signaling in the GABA synapses of medium spiny neurons. Phasic 2-AG-mediated short-term plasticity was also blunted, but this did not impact CB1R function or expression, indicating that the *FABP5* gene plays a role in central excitatory and inhibitory synapse signaling [28]. While not much is known regarding how the eCB levels influence the metabolism of nicotine, it is clear that nicotine influences the eCB levels which, in turn, enhances the reinforcing effects of the drug.

Our previous work showed a significant decrease in ethanol consumption among mice treated with an inhibitor of FABPs (SBFI26). Specifically, male and female mice treated with SBFI26 consumed 24% and 42% less compared with their *FABP5*^+/+^ counterparts receiving the vehicle, respectively. This supports the interrelationship between nicotine, cannabis, and ethanol [45]. While this seems paradoxical, it suggests that the reduction in FABPs can result in a blunted response in the pre-neuronal release of DA, thereby reducing the ethanol-induced euphoria followed by the attenuation of ethanol-seeking behavior.

To date, however, few studies have examined the impact of the co-exposure of cannabinoids and nicotine on locomotor activity. The previously referenced work by Merritt and colleagues reported no difference in the locomotor activity of C57BL/6J mice following nicotine injections of 0.1 mg/kg compared to saline injections [24]. These data support our current findings, as we did not observe a significant effect on the locomotor activity after the sc injections of 0.1 mg/kg nicotine in the case of either genotype. Based on this, it is likely that the increased nicotine preference, which is believed to be CB1R-dependent, is not influenced by locomotor activity.

Future studies should aim to explore the effects of *FABP5*^−/−^ on nicotine self-administration and withdrawal. Blocking CB1R activation via antagonists or inverse agonists has been shown to decrease nicotine-seeking behavior and self-administration and lessen nicotine withdrawal symptoms [14,24,46]. Therefore, it is likely that *FABP5*^−/−^ mice would display opposite effects due to their heightened AEA levels and CB1R activation in the mesolimbic reward pathway. While this enhanced nicotine preference appears to be CB1R-dependent, researchers should examine potential treatment methods for nicotine and other substances of abuse. For example, inhibiting the activity of CB1R has been explored for Δ9-tetrahydrocannabinol (THC), but the long-term blocking of CB1R would disinhibit GABA signaling. As a result, the neuronal release of DA would be reduced, which may, in turn, lead to enhanced substance use and abuse [47]. It is crucial to understand how both nicotine and THC interact with the eCB system, as tobacco use commonly follows or coincides with cannabis use [48]. Additionally, further studies should be conducted to determine the long-term effects of heightened AEA levels on nicotine metabolism in *FABP5*^−/−^ mice. The observed increase in nicotine-seeking behavior may be due to differences in metabolism, but this has not yet been confirmed. While our study focuses on the influence of the eCB system on nicotine-seeking behavior, it is important to examine other mechanisms involved in nicotine use in order to determine the best potential treatment methods. Previous studies found that tobacco smoke exposure leads to nicotine dependence in rats, which resulted in increased alpha-7 nicotinic acetylcholine receptors (nAChR) in the hippocampus and was correlated with increased somatic symptoms of withdrawal [49]. Additionally, corticotropin-releasing-factor (CRF)-like peptides have been linked to prolonged symptoms of withdrawal from cannabis, alcohol, and tobacco. While no studies have observed a direct effect of nicotine withdrawal on CRF production, the chronic administration of nicotine may alter the sensitivity of CRF-like peptides to their receptors [50]. When examining both nicotine self-administration and withdrawal symptoms in future studies, it is important to consider not only the eCBs but also changes in the nAChRs and CRF-like peptides.

While our study does point to the potential importance of the *FABP5* gene for nicotine-seeking behavior, it is not without limitations. Specifically, we do not know the exact effect of the eCB levels on nicotine metabolism. Higher eCB levels may slow down the metabolism of nicotine which may, therefore, potentiate its effects, but this is unknown. Future studies should aim to examine the pharmacological metabolism of nicotine in *FABP5*^−/−^ mice or other genetic models which display enhanced eCB levels.

Our findings support the conclusion that the eCB levels have an important influence on nicotine preference. We showed, for the first time, that the global deletion of *FABP5* potentiates the reinforcing aspects of low doses of nicotine, as measured by CPP. Future research should aim to directly examine the eCB levels in genetic models of *FABP5*^−/−^ in response to nicotine and the influence of the eCB levels on nicotine metabolism in order to confirm these notions.

## Data Availability

Not applicable.

## References

[1] Lariscy JT; Hummer R; Rogers R Cigarette Smoking and All-Cause and Cause-Specific Adult Mortality in the United States. Demography 2018, 55, 1855–1885. 30232778 10.1007/s13524-018-0707-2 PMC6219821

[2] Benowitz NL Pharmacology of Nicotine: Addiction, Smoking-Induced Disease, and Therapeutics. Annu. Rev. Pharmacol. Toxicol. 2009, 49, 57–71. 18834313 10.1146/annurev.pharmtox.48.113006.094742 PMC2946180

[3] Zhang T; Zhang L; Liang Y; Siapas AG; Zhou F-M; Dani JA Dopamine Signaling Differences in the Nucleus Accumbens and Dorsal Striatum Exploited by Nicotine. J. Neurosci 2009, 29, 4035. 19339599 10.1523/JNEUROSCI.0261-09.2009 PMC2743099

[4] Schultz W Dopamine signals for reward value and risk: Basic and recent data. Behav. Brain Funct 2010, 6, 24. 20416052 10.1186/1744-9081-6-24 PMC2876988

[5] Febo M; Blum K; Badgaiyan RD; Baron D; Thanos PK; Colon-Perez LM; Demotrovics Z; Gold MS Dopamine homeostasis: Brain functional connectivity in reward deficiency syndrome. Front. Biosci 2017, 22, 669–691. 10.2741/4509 27814639

[6] Melis M; Muntoni A; Pistis M Endocannabinoids and the Processing of Value-Related Signals. Front. Pharmacol 2012, 3, 7. 22347186 10.3389/fphar.2012.00007 PMC3270484

[7] Parsons LH; Hurd Y Endocannabinoid signalling in reward and addiction. Nat. Rev. Neurosci 2015, 16, 579–594. 26373473 10.1038/nrn4004 PMC4652927

[8] Murillo-Rodríguez E; Vázquez E; Millán-Aldaco D; Palomero-Rivero M; Drucker-Colin R Effects of the fatty acid amide hydrolase inhibitor URB597 on the sleep-wake cycle, c-Fos expression and dopamine levels of the rat. Eur. J. Pharmacol 2007, 562, 82–91. 17336288 10.1016/j.ejphar.2007.01.076

[9] Zhang L-Y; Zhou Y-Q; Yu Z-P; Zhang X-Q; Shi J; Shen H-W Restoring glutamate homeostasis in the nucleus accumbens via endocannabinoid-mimetic drug prevents relapse to cocaine seeking behavior in rats. Neuropsychopharmacology 2021, 46, 970–981. 33514875 10.1038/s41386-021-00955-1 PMC8115336

[10] Gunduz-Cinar O; MacPherson KP; Cinar R; Gamble-George J; Sugden K; Williams B; Godlewski G; Ramikie TS; Gorka AX; Alapafuja SO; Convergent translational evidence of a role for anandamide in amygdala-mediated fear extinction, threat processing and stress-reactivity. Mol. Psychiatry 2013, 18, 813–823. 22688188 10.1038/mp.2012.72 PMC3549323

[11] Garani R; Watts J; Mizrahi R Endocannabinoid system in psychotic and mood disorders, a review of human studies. Prog. Neuro-Psychopharmacol. Biol. Psychiatry 2021, 106, 110096. 10.1016/j.pnpbp.2020.110096 PMC8582009 32898588

[12] Blum K; Khalsa J; Cadet JL; Baron D; Bowirrat A; Boyett B; Lott L; Brewer R; Gondré-Lewis M; Bunt G; Cannabis-Induced Hypodopaminergic Anhedonia and Cognitive Decline in Humans: Embracing Putative Induction of Dopamine Homeostasis. Front. Psychiatry 2021, 12, 623403. 33868044 10.3389/fpsyt.2021.623403 PMC8044913

[13] Le Foll B; Goldberg S Rimonabant, a CB1 antagonist, blocks nicotine-conditioned place preferences. Neuroreport 2004, 15, 2139–2143. 15486497 10.1097/00001756-200409150-00028

[14] Cohen C; Perrault G; Griebel G; Soubrié P Nicotine-associated cues maintain nicotine-seeking behavior in rats several weeks after nicotine withdrawal: Reversal by the cannabinoid (CB1) receptor antagonist, rimonabant (SR141716). Neuropsychopharmacology 2005, 30, 145–155. 15292905 10.1038/sj.npp.1300541

[15] Le Foll B; Perrault G; Griebel G; Soubrié P Blocking cannabinoid CB1 receptors for the treatment of nicotine dependence: Insights from pre-clinical and clinical studies. Addict. Biol 2008, 13, 239–252. 18482433 10.1111/j.1369-1600.2008.00113.x PMC2752688

[16] Cheer JF; Wassum KM; Sombers LA; Heien MLAV; Ariansen JL; Aragona BJ; Phillips PEM; Wightman RM Phasic dopamine release evoked by abused substances requires cannabinoid receptor activation. J. Neurosci 2007, 27, 791–795. 17251418 10.1523/JNEUROSCI.4152-06.2007 PMC6672925

[17] Cohen C; Perrault G; Voltz C; Steinberg R; Soubrié P SR141716, a central cannabinoid (CB(1)) receptor antagonist, blocks the motivational and dopamine-releasing effects of nicotine in rats. Behav. Pharmacol 2002, 13, 451–463. 12394421 10.1097/00008877-200209000-00018

[18] Grieder TE; George O; Tan H; George SR; Le Foll B; Laviolette SR; van der Kooy D Phasic D1 and tonic D2 dopamine receptor signaling double dissociate the motivational effects of acute nicotine and chronic nicotine withdrawal. Proc. Natl. Acad. Sci. USA 2012, 109, 3101–3106. 22308372 10.1073/pnas.1114422109 PMC3286981

[19] Forget B; Coen K; Le Foll B Inhibition of fatty acid amide hydrolase reduces reinstatement of nicotine seeking but not break point for nicotine self-administration—Comparison with CB(1) receptor blockade. Psychopharmacology 2009, 205, 613–624. 19484221 10.1007/s00213-009-1569-5

[20] Kandel ER; Kandel DB A molecular basis for nicotine as a gateway drug. N. Engl. J. Med 2014, 371, 932–943. 25184865 10.1056/NEJMsa1405092 PMC4353486

[21] Rodríguez de Fonseca F; del Arco I; Bermudez-Silva FJ; Bilbao A; Cippitelli A; Navarro M The endocannabinoid system: Physiology and pharmacology. Alcohol Alcohol. 2005, 40, 2–14. 15550444 10.1093/alcalc/agh110

[22] Devane WA; Hanuš L; Breuer A; Pertwee RG; Stevenson LA; Griffin G; Gibson D; Mandelbaum A; Etinger A; Mechoulam R Isolation and structure of a brain constituent that binds to the cannabinoid receptor. Science 1992, 258, 1946–1949. 1470919 10.1126/science.1470919

[23] Cravatt BF; Giang DK; Mayfield SP; Boger DL; Lerner RA; Gilula NB Molecular characterization of an enzyme that degrades neuromodulatory fatty-acid amides. Nature 1996, 384, 83–87. 8900284 10.1038/384083a0

[24] Merritt LL; Martin BR; Walters C; Lichtman AH; Damaj MI The endogenous cannabinoid system modulates nicotine reward and dependence. J. Pharmacol. Exp. Ther 2008, 326, 483–492. 18451315 10.1124/jpet.108.138321 PMC2746999

[25] Pavon FJ; Serrano A; Sidhpura N; Polis I; Stouffer D; de Fonseca FR; Cravatt BF; Martin-Fardon R; Parsons LH Fatty acid amide hydrolase (FAAH) inactivation confers enhanced sensitivity to nicotine-induced dopamine release in the mouse nucleus accumbens. Addict. Biol 2018, 23, 723–734. 28660730 10.1111/adb.12531 PMC5747548

[26] Kaczocha M; Glaser S; Deutsch D Identification of intracellular carriers for the endocannabinoid anandamide. Proc. Natl. Acad. Sci. USA 2009, 106, 6375–6380. 19307565 10.1073/pnas.0901515106 PMC2669397

[27] Kaczocha M; Glaser ST; Maher T; Clavin B; Hamilton J; Joseph O; Rebecchi M; Puopolo M; Owada Y; Thanos PK Fatty acid binding protein deletion suppresses inflammatory pain through endocannabinoid/N-acylethanolamine-dependent mechanisms. Mol. Pain 2015, 11, 52. 26311517 10.1186/s12990-015-0056-8 PMC4551694

[28] Fauzan M; Oubraim S; Yu M; Glaser ST; Kaczocha M; Haj-Dahmane S Fatty Acid-Binding Protein 5 Modulates Brain Endocannabinoid Tone and Retrograde Signaling in the Striatum. Front. Cell. Neurosci 2022, 16, 936939. 35875351 10.3389/fncel.2022.936939 PMC9302024

[29] Yu S; Levi L; Casadesus G; Kunos G; Noy N Fatty acid-binding protein 5 (FABP5) regulates cognitive function both by decreasing anandamide levels and by activating the nuclear receptor peroxisome proliferator-activated receptor β/δ (PPARβ/δ) in the brain. J. Biol. Chem 2014, 289, 12748–12758. 24644281 10.1074/jbc.M114.559062 PMC4007463

[30] Buczynski MW; Polis I; Parsons L The volitional nature of nicotine exposure alters anandamide and oleoylethanolamide levels in the ventral tegmental area. Neuropsychopharmacology 2013, 38, 574–584. 23169348 10.1038/npp.2012.210 PMC3572454

[31] Maeda K; Uysal KT; Makowski L; Görgün CZ; Atsumi G; Parker RA; Brüning J; Hertzel AV; Bernlohr DA; Hotamisligil GS Role of the fatty acid binding protein mal1 in obesity and insulin resistance. Diabetes 2003, 52, 300–307. 12540600 10.2337/diabetes.52.2.300 PMC4027060

[32] Thanos K; Bermeo C; Wang G-J; Volkowa ND D-cycloserine accelerates the extinction of cocaine-induced conditioned place preference in C57bL/c mice. Behav. Brain Res 2009, 199, 345–349. 19152811 10.1016/j.bbr.2008.12.025 PMC2653598

[33] Thanos K; Malave L; Delis F; Mangine P; Kane K; Grunseich A; Vitale M; Greengard P; Volkow ND Knockout of p11 attenuates the acquisition and reinstatement of cocaine conditioned place preference in male but not in female mice. Synapse 2016, 70, 293–301. 26990537 10.1002/syn.21904

[34] Thanos PK; Bermeo C; Rubinstein M; Suchland KL; Wang GJ; Grandy DK; Volkow ND Conditioned place preference and locomotor activity in response to methylphenidate, amphetamine and cocaine in mice lacking dopamine D4 receptors. J. Psychopharmacol 2010, 24, 897–904. 19282420 10.1177/0269881109102613 PMC2878389

[35] Hamilton J; Marion M; Figueiredo A; Clavin BH; Deutsch D; Kaczocha M; Haj-Dahmane S; Thanos PK Fatty acid binding protein deletion prevents stress-induced preference for cocaine and dampens stress-induced corticosterone levels. Synapse 2018, 72, e22031. 29457656 10.1002/syn.22031 PMC6341999

[36] Ananth M; Hetelekides EM; Hamilton J; Thanos PK Dopamine D4 receptor gene expression plays important role in extinction and reinstatement of cocaine-seeking behavior in mice. Behav. Brain Res 2019, 365, 1–6. 30797855 10.1016/j.bbr.2019.02.036

[37] Cravatt BF; Demarest K; Patricelli MP; Bracey MH; Giang DK; Martin BR; Aron H Lichtman Supersensitivity to anandamide and enhanced endogenous cannabinoid signaling in mice lacking fatty acid amide hydrolase. Proc. Natl. Acad. Sci. USA 2001, 98, 9371. 11470906 10.1073/pnas.161191698 PMC55427

[38] Haj-Dahmane S; Shen R Regulation of plasticity of glutamate synapses by endocannabinoids and the cyclic-AMP/protein kinase A pathway in midbrain dopamine neurons. J. Physiol 2010, 588, 2589–2604. 20498231 10.1113/jphysiol.2010.190066 PMC2916990

[39] Mansvelder HD; Mertz M; Role L Nicotinic modulation of synaptic transmission and plasticity in cortico-limbic circuits. Semin. Cell. Dev. Biol 2009, 20, 432–440. 19560048 10.1016/j.semcdb.2009.01.007 PMC2742626

[40] Oleson EB; Beckert MV; Morra JT; Lansink CS; Cachope R; Abdullah RA; Loriaux AL; Schetters D; Pattij T; Roitman MF; Endocannabinoids shape accumbal encoding of cue-motivated behavior via CB1 receptor activation in the ventral tegmentum. Neuron 2012, 73, 360–373. 22284189 10.1016/j.neuron.2011.11.018 PMC3269037

[41] Lupica CR; Riegel AC Endocannabinoid release from midbrain dopamine neurons: A potential substrate for cannabinoid receptor antagonist treatment of addiction. Neuropharmacology 2005, 48, 1105–1116. 15878779 10.1016/j.neuropharm.2005.03.016

[42] Szabo B; Siemes S; Wallmichrath I Inhibition of GABAergic neurotransmission in the ventral tegmental area by cannabinoids. Eur. J. Neurosci 2002, 15, 2057–2061. 12099913 10.1046/j.1460-9568.2002.02041.x

[43] Melis M; Pistis M; Perra S; Muntoni AL; Pillolla G; Gessa GL Endocannabinoids mediate presynaptic inhibition of glutamatergic transmission in rat ventral tegmental area dopamine neurons through activation of CB1 receptors. J. Neurosci 2004, 24, 53–62. 14715937 10.1523/JNEUROSCI.4503-03.2004 PMC6729571

[44] Melis M; Pillolla G; Luchicchi A; Muntoni AL; Yasar S; Goldberg SR; Pistis M Endogenous fatty acid ethanolamides suppress nicotine-induced activation of mesolimbic dopamine neurons through nuclear receptors. J. Neurosci 2008, 28, 13985–13994. 19091987 10.1523/JNEUROSCI.3221-08.2008 PMC3169176

[45] Figueiredo A; Hamilton J; Marion M; Blum K; Kaczocha M; Haj-Dahmane S; Deutsch D; Thanos PK Pharmacological Inhibition of Brain Fatty Acid Binding Protein Reduces Ethanol Consumption in Mice. J. Reward Defic. Syndr. Addict. Sci 2017, 3, 21–27. 29367955 PMC5777574

[46] Cohen C; Kodas E; Griebel G CB1 receptor antagonists for the treatment of nicotine addiction. Pharmacol. Biochem. Behav 2005, 81, 387–395. 15935455 10.1016/j.pbb.2005.01.024

[47] Blum K; Oscar-Berman M; Braverman ER; Febo M; Li M; Gold MS Enhancing Brain Pregnenolone May Protect Cannabis Intoxication but Should Not Be Considered as an Anti-addiction Therapeutic: Hypothesizing Dopaminergic Blockade and Promoting Anti-Reward. J. Reward Defic. Syndr 2015, 1, 20–23. 26306328 10.17756/jrds.2015-005 PMC4545660

[48] Tullis LM; Dupont R; Frost-Pineda K; Gold MS Marijuana and tobacco: A major connection? J. Addict. Dis 2003, 22, 51–62. 14621344 10.1300/J069v22n03_05

[49] Small E; Shah HP; Davenport JJ; Geier JE; Yavarovich KR; Yamada H; Sabarinath SN; Derendorf H; Pauly JR; Gold MS; Tobacco smoke exposure induces nicotine dependence in rats. Psychopharmacology 2010, 208, 143–158. 19936715 10.1007/s00213-009-1716-z PMC3586198

[50] Bruijnzeel AW; Gold MS The role of corticotropin-releasing factor-like peptides in cannabis, nicotine, and alcohol dependence. Brain Res. Rev 2005, 49, 505–528. 16269317 10.1016/j.brainresrev.2005.01.007

